# Diagnosis of Gall-Stones

**Published:** 1903-05-30

**Authors:** 


### DIAGNOSIS OF GALL-STONES.

In dealing with this important question Dr. Murphy [1] confines his attention for the most part to those types of the affection where the classic symptoms of colic, nausea and vomiting, local tenderness and icterus, are one or more of them wanting. His observations are, in the main, based on his own experience of 334 cases of gall-bladder and liver disease. Taking the symptoms he classifies their different varieties. Pain may be of four kinds :—(1) the acute inflammatory type which occurs in virulent infections of the gall-bladder and is accompanied by rigidity of the overlying muscles. (2) The aching type which is not accompanied by rigidity of protecting muscles. The most characteristic reaction in this sort of pain is that elicited by pressure applied beneath the right costal arch when the patient makes an effort at deep inspiration ; as soon as the sensitive gall-bladder reaches the examining fingers inspiration is arrested with a jerk. Dr. Murphy has never found this sign absent in a calculous or infectious case of gall-bladder, or duct disease. (3) The referred ache or pain which accompanies either of the preceding types. In obstructions to the pelvis of the gall-bladder, or cystic duct, the pain is referred on an average in seven cases out of ten to the right subscapular region, in one to the left subscapular region, in the other two to some other region of the thorax. In all there is a local pain in the gall-bladder region as well as the referred pain. These figures are based upon repeated soundings after cholecystostomy. (4) Colic. This is never due to distension of the gall-bladder, and never due to the retention of bile or mucus under pressure. It only occurs during the *transit* of a stone or other foreign body along the duct. If the foreign body become stationary in the duct beyond a few hours the colic will cease. It is probably due to muscular contraction in the duct wall, and certainly does not depend upon inflammation of the gall-bladder. Colic does not occur in blocking of the duct from catarrhal or infective swelling of its mucosa, nor from obstruction due to new growth, cicatricial occlusions or flexions. It is therefore characteristic of the presence of a foreign body in one of the ducts. Colic occurs when a stone moves backwards in the cystic duct into the gall-bladder as sometimes occurs after cholecystostomy. Hypersensitiveness of the gall-bladder is present in all varieties of infection and calculous obstruction, but is absent in blocking due to any other cause, *e.g.* new growth. Reflex vomiting may occur and may even be pronounced in cases where there is not much pain and no colic. In these instances the symptom is very misleading, and may lead to a diagnosis of pyloric obstruction or some other gastric affection. The temperature in biliary infection is almost pathognomonic, and is peculiar in the angularity of the chart and the long duration of the complete intermissions.

In 80 per cent. of all cases of cholelithiasis with jaundice, the gall-bladder is contracted or normal in size. In the remaining 20 per cent., in which the gall-bladder is enlarged, the distension is due to lodgment of a stone in the cystic or common duct. Where there is obstruction of the biliary passages due to other causes than stone, there is a recognisable increase in the size of the gall-bladder in 90 per cent. Courvoisier, who worked out these numbers, laid down the following law : " In 80 per cent. of the cases of obstruction of the common duct due to stone there is contraction of the gall-bladder while in 90 per cent. of the cases of enlargement of the gall-bladder the blocking is due to other causes than stone." In 86 per cent. of Dr. Murphy's cases of cholelithiasis jaundice was absent, and a history of attacks of jaundice could not be obtained. When jaundice is present its intensity depends upon the completeness or otherwise of the obstruction. In malignant occlusion of the bile passages jaundice is continuous throughout the entire course of the disease without a remission. In calculous disease, on the other hand, jaundice may be remittent, and for this reason that the blocking of the duct is not solely due to the size of an impacted stone, but in part is the result of inflammatory swelling of the mucous membrane of the duct. With rest and time this œdema of the mucosa may subside sufficiently to allow bile to pass, but it is likely to recur so long as the stone remains in the canal, and it usually does recur before the jaundice has time to completely disappear. The jaundice is thus remittent, but not intermittent.

Intermittent jaundice may be met with in cholelithiasis, and is due either to successive stones passing through the common duct, or to the lodgement of a stone in the cystic duct setting up inflammation which may extend to and cause temporary blocking of the hepatic and common ducts. In the relation of jaundice to pain this practical rule may be formulated : (1) That jaundice due to gall-stones is always preceded by colic ; (2) that jaundice due to malignant disease, or catarrh of the ducts accompanied by or due to infection, is never preceded by colic. Stones may be present in the gall-bladder without being recognised during life. Post-mortem records show that gall-stones are found to be present in from 6·94 per cent. to 12·2 per cent. of all necropsies. There is good reason to suppose that symptoms do not occur unless (1) a stone engages the neck of the gall-bladder or passes down the duct, or (2), the gall-bladder becomes infected.

[1] Medical News, May 2.

